# Parents' knowledge and attitudes towards extracorporeal membrane oxygenation and their post-traumatic stress symptoms

**DOI:** 10.1038/s41598-024-60880-3

**Published:** 2024-05-10

**Authors:** Yuyan Sun, Huanhuan Wang, Yingying Wu, Leng Luo, Caixiao Shi

**Affiliations:** 1 Surgical Intensive Care Unit, Henan Children′s Hospital Zhengzhou Children′s Hospital, Henan, 450018 China; 2 Rehabilitation Department, Henan Children′s Hospital Zhengzhou Children′s Hospital, Henan, 450053 China; 3 Nursing Department, Henan Children′s Hospital Zhengzhou Children′s Hospital, Henan, 450018 China

**Keywords:** Knowledge, Attitude, Practice, Extracorporeal membrane oxygenation (ECMO), Post-traumatic stress symptoms (PTSS), Parents, Diseases, Risk factors

## Abstract

This cross-sectional study, conducted between January 2020 and July 2023, aimed to assess the knowledge, attitude, and post-traumatic stress symptoms (PTSS) among parents with children undergoing extracorporeal membrane oxygenation (ECMO) treatment. Out of 201 valid questionnaires collected, the median knowledge score was 3.00, the mean attitude score was 27.00 ± 3.20, and the mean PTSS score was 3.50 ± 1.54. Logistic regression identified associations between PTSS and parents with lower education levels, particularly junior high school and high school/technical secondary school education, as well as those occupied as housewives. Structural equation modeling highlighted direct effects, such as the impact of residence on education, education on employment status, and associations between knowledge, attitude, PTSS, employment status, monthly income, and parental demographics. The findings indicated inadequate knowledge and suboptimal attitudes among parents, especially those with lower education levels, emphasizing the need for educational resources. Furthermore, addressing parental PTSS through psychosocial support and screening was deemed essential, providing valuable insights for tailored interventions in this context.

## Introduction

Extracorporeal Membrane Oxygenation (ECMO) has seen significant growth in its applications for treating severe acute respiratory failure (Veno-Venous ECMO) and circulatory failure (Veno-Arterial ECMO)^1^. This expansion is largely attributed to advancements in ECMO technology and increased clinical experience^2^. The number of pediatric patients benefiting from ECMO support has also been on the rise, with the Extracorporeal Life Support Organization (ELSO) reporting 3249 infants and children receiving ECMO in 2015^3^. Notably, neonatal and pediatric ECMO cases constitute nearly 50% and 25% of the total ECMO interventions, respectively, according to ELSO's registry data^4^. Symptoms of post-traumatic stress disorder (PTSD) emerge subsequent to the experience of a significantly distressing event. These symptoms collectively manifest as a syndrome characterized by heightened arousal and agitation, recurrent re-experiencing of the traumatic event, and deliberate avoidance of stimuli associated with the trauma^5^. Concurrently, it is essential to consider the psychological ramifications, as Post-traumatic stress symptoms (PTSS) can result from traumatic experiences, and a child's critical illness can be profoundly distressing for parents, potentially leading to symptoms of Acute Stress Disorder (ASD), PTSS, and full-fledged PTSS within the family unit, highlighting the critical need for comprehensive research and support in this context^6,7^.

A Knowledge, Attitude, and Practices (KAP) survey is a diagnostic research methodology used to assess a specific group's comprehension, viewpoints, and actions regarding a particular subject matter, encompassing their knowledge, attitudes, and behavioral patterns related to the designated topic^8–10^. Given the urgent and highly stressful nature of ECMO therapy, which can have lasting psychological effects on patient families, and the limited exploration of the psychological and emotional impact of ECMO in existing research, examining parents' knowledge and attitudes towards ECMO is important. This study may help medical teams better understand the needs of ECMO patient families, improve communication, provide appropriate support, and guide the development of interventions for their psychological well-being, while also offering insights for further research in related areas.

Healthcare providers should be aware of the elevated rates of post-traumatic stress experienced by parents of children who have survived critical illness^11^. Implementing routine screening for post-traumatic stress symptoms and offering support resources can facilitate early intervention. A longitudinal study^12^ found that family members primarily relied on problem-focused coping strategies, with anxiety symptoms peaking during treatment initiation but gradually decreasing over time, along with reductions in depression and PTSD symptoms. However, Health-Related Quality of Life (HRQOL) remained significantly impaired, indicating persistent emotional distress despite symptom improvement. Similarly, a retrospective analysis^13^ identified a high prevalence of PTSD symptoms among adult survivors of veno-arterial ECMO (VA-ECMO) support, particularly in patients with longer ICU stays requiring sedation and mechanical ventilation, underscoring the potential psychological impact of prolonged critical care treatment. Despite its significance, there has been a lack of KAP studies in this area. Therefore, this study aimed to investigate knowledge, attitude, and parental PTSS among parents whose children undergoing ECMO treatment.

## Methods

### Study design and participants

This cross-sectional study was conducted in five tertiary-level Grade A hospitals in Henan Province between January 2020 and July 2023. The study encompassed parents of children aged 0–12 undergoing Extracorporeal Membrane Oxygenation (ECMO) treatment, with specific inclusion and exclusion criteria set forth. Inclusion criteria comprised parents meeting the following conditions: (1) Having children aged 0–12 undergoing ECMO therapy, (2) Demonstrating adequate literacy and lacking communication barriers, and (3) Serving as the primary caregivers for their children under regular circumstances. Exclusion criteria, on the other hand, entailed the following conditions: (1) A history of mental illness or cognitive impairments, (2) Concurrently caring for another critically ill child at home, (3) Parental status for children undergoing ECMO for transplantation, and (4) Parental status for children afflicted with malignant tumors or severe infectious diseases.

This study received ethical approval from the Ethics Committee of Henan Children's Hospital and obtained informed consent from the study participants.

### Procedures

The questionnaire was developed based on the consensus of ECMO application experts in the clinical field, covering four key dimensions. To refine the questionnaire, input was gathered from 11 experts in pediatric critical care to eliminate redundancy, enhance clarity, and ensure conciseness. A pilot study involving 31 participants was conducted, resulting in an overall Cronbach's α coefficient of 0.812, indicating robust internal consistency across the overall questionnaire.

The final questionnaire comprised the following sections: Demographic information about the participants, including age, gender, residence type, education level, employment status, income level, and more. The Knowledge dimension consisted of 9 questions, with responses scored as “very familiar” (2 points), “heard of it” (1 point), or “unfamiliar” (0 points). The Attitude dimension included 8 questions, utilizing a five-point Likert scale ranging from “very positive” (5 points) to “very negative” (1 point). The PTSS-related questionnaire contained 5 questions, with responses scored as option “a” (1 point) or option “b” (0 points), where a score above 2 indicated a potential PTSS condition^14^. Higher scores indicated better knowledge and more positive attitudes among the survey respondents.

Before official distribution, a small-scale pilot test involving 31 responses was conducted, resulting in a robust Cronbach's α coefficient of 0.766, indicative of strong internal consistency. Contact with hospitals was established through various means, including WeChat, phone calls, and email, with surveys administered to all eligible participants within the hospital settings. The distribution of questionnaires was overseen by eight research assistants who formed a survey team that underwent standardized training. These assistants provided consistent instructions, obtained informed consent, clarified survey procedures, guidelines, and confidentiality principles, and subsequently administered questionnaires in both digital and paper formats. For participants less familiar with electronic devices, paper questionnaires were provided, while others accessed the survey through online QR codes. The process was closely monitored by research assistants to discourage hasty completion. Participants independently completed the questionnaire if they understood the questions; otherwise, surveyors conducted face-to-face interviews, recording responses based on verbal input. After questionnaire completion, a rigorous examination of collected questionnaires was carried out. Data were entered twice by two individuals, with 20% of randomly selected samples subjected to re-entry to ensure data consistency and research data accuracy.

### Statistical analysis

Statistical analysis was conducted using Stata 17.0 (Stata Corporation, Junior college Station, TX, USA). Quantitative variables were described using mean ± standard deviation (SD), and between-group comparisons were performed using t-tests or analysis of variance (ANOVA). Categorical variables were presented as n (%). Pearson correlation analysis was employed to assess the correlations between knowledge, attitude, and practice scores. Univariate variables with P < 0.05 were enrolled in multivariate regression. Hypotheses were validated using structural equation modeling (SEM). Hypotheses were validated using structural equation modeling (SEM). The hypotheses posited that Knowledge exerts a direct influence on Attitude and PTSS, Attitude directly impacts PTSS, and that residence, education, employment status, and monthly income directly affect Knowledge, Attitude, and PTSS. Two-sided P < 0.05 were considered statistically significant in this study.

### Ethics approval and consent to participate

This work has been carried out in accordance with the Declaration of Helsinki (2000) of the World Medical Association. This study received ethical approval from the Ethics Committee of Henan Children's Hospital (2023-K-150) and obtained informed consent from the study participants.

## Results

Initially, a total of 212 cases were collected in this study. The following cases were deleted: (1) 1 case with the age filled in as “330” and 1 case with the age filled in as “250”; (2) 8 cases in which the number of children in the family was filled in as 0 or none, but continued to answer the questions about children; (3) 1 case in which the number of children was filled in as “21”; the final valid questionnaire was 201 cases. Among these, 111 (55.22%) were female, with a mean age of 32 (range: 20–56) years, and most had one child. Additionally, 77 (38.31%) had completed junior college or undergraduate education, while 147 (73.13%) were employed. Of the participants, 75 (37.31%) indicated that their children required ECMO due to acute respiratory distress, and 183 (91.04%) had medical insurance coverage.

The median knowledge score was 3.00 (0–18) (possible range: 0–18), the mean attitude score was 27.00 ± 3.20 (possible range: 8—40), and the mean PTSS score was 3.50 ± 1.54 (possible range: 0—5). Parents with different genders were more likely to have different PTSS score (P = 0.050). Moreover, the knowledge, attitude, PTSS scores varied from those with different residence (P < 0.001, P < 0.001, and P = 0.004, separately), education (P < 0.001, P < 0.001, and P < 0.001, separately), employment status (P < 0.001, P < 0.001, and P = 0.007, separately), and monthly per capita income (P < 0.001, P < 0.001, and P = 0.010, separately) (Table 1).

**Table 1:** Baseline characteristics and KAP scores.

Variables	N (%)	Knowledge, Median (range)	P	Attitude, mean ± SD	P	PTSS, mean ± SD	P
n = 201							
Total score		3.00 (0.00–18.00)		27.00 ± 3.20		3.50 ± 1.54	
Gender			0.166		0.069		0.050
Male	90 (44.78)	3.00 (0.00–18.00)		27.46 ± 3.33		3.27 ± 1.53	
Female	111 (55.22)	3.00 (0.00–18.00)		26.63 ± 3.06		3.69 ± 1.53	
Age, years	32 (20–56)						
Number of children	1 (1–4)						
Residence			< 0.001		< 0.001		0.004
Rural	92 (45.77)	2.00 (0.00–18.00)		26.01 ± 2.82		3.76 ± 1.55	
Urban	98 (48.76)	4.00 (0.00–18.00)		27.74 ± 3.29		3.41 ± 1.41	
Suburban	11 (5.47)	4.00 (0.00–9.00)		28.64 ± 3.11		2.18 ± 1.94	
Education			< 0.001		< 0.001		< 0.001
Primary school or below	10 (4.98)	1.00 (0.00–16.00)		24.40 ± 3.37		4.70 ± 0.67	
Junior high school	31 (15.42)	0.00 (0.00–18.00)		25.10 ± 2.70		3.97 ± 1.54	
High school/Technical secondary school	57 (28.36)	2.00 (0.00–9.00)		25.93 ± 2.28		3.96 ± 0.98	
Junior college/Undergraduate	77 (38.31)	5.00 (0.00–18.00)		27.74 ± 3.12		3.10 ± 1.73	
Postgraduate and above	26 (12.94)	8.50 (0.00–18.00)		30.42 ± 2.02		2.65 ± 1.47	
Employment status			< 0.001		< 0.001		0.007
Employed	147 (73.13)	3.00 (0.00–18.00)		27.41 ± 3.18		3.46 ± 1.43	
Unemployed	21 (10.45)	1.00 (0.00–4.00)		24.71 ± 2.24		4.38 ± 1.16	
Retired	2 (1.00)	4.00 (3.00–5.00)		23.50 ± 0.71		2.50 ± 0.71	
Self-employed	11 (5.47)	2.00 (0.00–7.00)		25.82 ± 1.89		4.09 ± 1.51	
Housewife	10 (4.98)	0.50 (0.00–9.00)		25.60 ± 2.22		2.70 ± 2.36	
Other	10 (4.98)	7.00 (0.00–18.00)		29.20 ± 4.08		2.60 ± 2.12	
Monthly per capita income, yuan			< 0.001		< 0.001		0.010
< 2000	21 (10.45)	1.00 (0.00–5.00)		24.38 ± 1.91		3.86 ± 1.77	
2000–5000	77 (38.31)	2.00 (0.00–18.00)		26.23 ± 2.88		3.61 ± 1.47	
5000–10,000	79 (39.30)	5.00 (0.00–18.00)		27.66 ± 3.13		3.62 ± 1.42	
10,000–20,000	21 (10.45)	9.00 (1.00–18.00)		29.76 ± 2.79		2.48 ± 1.72	
> 20,000	3 (1.49)	5.00 (0.00–14.00)		28.33 ± 4.04		2.33 ± 0.58	
Cause of child's ECMO application			0.881		0.789		0.361
Acute Myocarditis	60 (29.85)	2.00 (0.00–18.00)		26.90 ± 3.25		3.70 ± 1.38	
Acute Respiratory Distress Syndrome	75 (37.31)	3.00 (0.00–18.00)		27.13 ± 3.26		3.33 ± 1.62	
Post-Cardiac Surgery	43 (21.39)	3.00 (0.00–16.00)		26.67 ± 3.18		3.67 ± 1.34	
Other	12 (11.44)	3.00 (0.00–18.00)		27.43 ± 3.04		3.22 ± 1.95	
Medical insurance			0.570		0.758		0.637
Yes	183 (91.04)	3.00 (0.00–18.00)		27.02 ± 3.26		3.49 ± 1.56	
No	18 (8.96)	2.50 (0.00–10.00)		26.78 ± 2.53		3.67 ± 1.37	

No more than 10% chose option “Very familiar” for all knowledge section questions. The three questions with the highest number of participants choosing the “Heard of it” option were “Extracorporeal Membrane Oxygenation, colloquially referred to as “ECMO” or “artificial lung,” is a medical emergency device.” (K1) with 51.74%, “ECMO, as an effective adjunct for diseased hearts, can buy valuable time for further diagnosis and treatment of the heart.” (K5) with 36.82%, and “Complications of ECMO include bleeding, infection, central nervous system complications, thrombosis, and more.” (K8) with 36.82%. On the contrary, the three questions with the highest number of participants choosing the “Unfamiliar” option were “Whether it is a cardiac or respiratory disease, not all cases require VA (venous to arterial).” (K6) with 71.14%, “Internationally, ECMO can also be used in infants when necessary, and its effectiveness in infants is superior to that in adults.” (K7) with 71.14%, and “Due to ECMO punctures and catheter placement, local vascular damage may occur, resulting in long-term narrowing of blood vessels, possibly requiring subsequent vascular dilation procedures.” (K9) with 69.15% (Table 2).

**Table 2:** Responses in various knowledge dimensions.

Item, n (%)	Very familiar	Heard of it	Unfamiliar
1. Extracorporeal Membrane Oxygenation, colloquially referred to as “ECMO” or “artificial lung,” is a medical emergency device	17 (8.46)	104 (51.74)	80 (39.80)
2. ECMO is a form of cardiopulmonary life support that works by withdrawing venous blood from the body, oxygenating it, and then pumping it back into the patient's venous or arterial system, serving as a replacement for the heart and lungs	13 (6.47)	72 (35.82)	116 (57.71)
3. ECMO can be used for various reasons leading to cardiac or respiratory arrest, severe cardiac failure, severe respiratory failure, and various conditions that critically threaten respiratory and circulatory function, such as severe asthma, drowning, etc	14 (6.97)	71 (35.32)	116 (57.71)
4. ECMO can temporarily replace a patient's cardiopulmonary function, alleviating the burden on the patient's heart and lungs, in situations such as respiratory failure, cardiac arrest, etc	19 (9.45)	66 (32.84)	116 (57.71)
5. ECMO, as an effective adjunct for diseased hearts, can buy valuable time for further diagnosis and treatment of the heart	20 (9.95)	74 (36.82)	107 (53.23)
6. Whether it is a cardiac or respiratory disease, not all cases require VA (venous to arterial)	9 (4.48)	49 (24.38)	143 (71.14)
7. Internationally, ECMO can also be used in infants when necessary, and its effectiveness in infants is superior to that in adults	9 (4.48)	49 (24.38)	143 (71.14)
8. Complications of ECMO include bleeding, infection, central nervous system complications, thrombosis, and more	14 (6.97)	74 (36.82)	113 (56.22)
9. Due to ECMO punctures and catheter placement, local vascular damage may occur, resulting in long-term narrowing of blood vessels, possibly requiring subsequent vascular dilation procedures	10 (4.98)	52 (25.87)	139 (69.15)

In the context of ECMO's potential to extend treatment time for children (A1), it is notable that a significant majority of patients agree (56.93%). Examining the belief that all eligible patients should opt for ECMO (A2), 6.97% of respondents strongly agree, and 30.85% agree. Intriguingly, despite ECMO's high cost (A3), 13.43% of patients strongly agree, and 40.80% agree that it is a worthwhile option. Regarding the emotional impact of ECMO (A4), 10.95% of patients strongly agree, and 42.29% agree that ECMO instills great hope in them and their families. Conversely, concerns about potential harm following ECMO application (A5) are voiced by 11.44% of patients who strongly agree and 43.78% who agree. The understanding of ECMO's therapeutic function (A6) reveals that 6.47% of patients strongly agree, and 31.84% agree that it replaces respiratory or cardiopulmonary function rather than curing diseases. Furthermore, ECMO is not a panacea (A7), with 11.94% strongly agreeing and 51.24% agreeing that it should not be mythicized. Lastly, sentiments of regret about ECMO's limited promotion (A8) are expressed by 5.97% of patients who strongly agree and 31.34% who agree (Table 3).

**Table 3:** Responses in various attitude dimensions.

Item, n (%)	Strongly agree	Agree	Neutral	Disagree	Strongly disagree
1. I believe ECMO can provide more treatment time for children	22 (10.95)	93 (46.27)	84 (41.79)	2 (1.00)	0 (0.00)
2. I believe all eligible patients should choose ECMO	14 (6.97)	62 (30.85)	98 (48.76)	23 (11.44)	4 (1.99)
3. I believe that despite its high cost, ECMO is worthwhile	27 (13.43)	82 (40.80)	79 (39.30)	13 (6.47)	0 (0.00)
4. I believe ECMO has given my family and me great hope	22 (10.95)	85 (42.29)	87 (43.28)	7 (3.48)	0 (0.00)
5. I am very concerned about potential harm after ECMO application	23 (11.44)	88 (43.78)	83 (41.29)	7 (3.48)	0 (0.00)
6. ECMO does not cure diseases; it merely replaces respiratory or cardiopulmonary function	13 (6.47)	64 (31.84)	117 (58.21)	7 (3.48)	0 (0.00)
7. ECMO is not a panacea, and its existence should not be mythicized	24 (11.94)	103 (51.24)	60 (29.85)	10 (4.98)	4 (1.99)
8. I feel regretful that ECMO is not widely promoted to help more people in need	12 (5.97)	63 (31.34)	111 (55.22)	14 (6.97)	1 (0.50)

Correlation analysis revealed significant positive associations between knowledge and attitude (r = 0.470, P < 0.001). Conversely, negative correlations were observed between knowledge and PTSS (r = −0.358, P < 0.001), as well as between attitude and PTSS (r = −0.323, P < 0.001) (Table 4).

**Table 4:** Correlation analysis of knowledge, attitude, and PTSD.

	Knowledge	Attitude	PTSD
Knowledge	1		
Attitude	0.470 (*P* < 0.001)	1	
PTSD	−0.358 (*P* < 0.001)	−0.323 (*P* < 0.001)	1

Multivariate logistic regression analysis showed that junior high school education (OR = 28.260, 95% CI: 2.469–323.427, P = 0.007), high school/technical secondary school education (OR = 15.573, 95% CI: 2.589–93.680, P = 0.003), and the occupation of being a housewife (OR = 0.054, 95% CI: 0.005–0.548, P = 0.014) were independently associated with PTSS (Table 5).

**Table 5:** Univariate and multivariate logistic regression analysis for PTSD.

Characteristics	Univariate		Multivariate	
	OR (95%CI)	P	OR (95%CI)	P
Knowledge dimension	0.849 (0.787–0.917)	< 0.001	0.933 (0.837–1.041)	0.213
Attitude dimension	0.790 (0.707–0.884)	< 0.001	0.912 (0.775–1.073)	0.267
Gender				
Male	0.568 (0.288–1.121)	0.103		
Female	ref			
Age	1.011 (0.964–1.060)	0.661		
Residence				
Rural	ref		ref	
Urban	0.673 (0.325–1.395)	0.287	2.768 (0.979–7.828)	0.055
Suburban	0.162 (0.044–0.601)	0.007	1.119 (0.230–5.453)	0.889
Education				
Primary school or below	–	0.999	–	0.999
Junior high school	6.750 (1.837–24.802)	0.004	28.260 (2.469–323.427)	0.007
High school/Technical secondary school	13.250 (3.705–47.385)	< 0.001	15.573 (2.589–93.680)	0.003
Junior college/Undergraduate	2.500 (1.002–6.236)	0.049	2.421 (0.809–7.251)	0.114
Postgraduate and above	ref		ref	
Employment status				
Employed	ref		ref	
Unemployed	5.345 (0.690–41.397)	0.109	0.787 (0.078–7.953)	0.839
Retired	0.267 (0.016–4.395)	0.356	0.058 (0.003–1.341)	0.076
Self-employed	0.329 (21.681)	0.357	0.844 (0.090–7.899)	0.882
Housewife	0.401 (0.106–1.509)	0.177	0.054 (0.005–0.548)	0.014
Other	0.267 (0.073–0.982)	0.047	0.279 (0.055–1.422)	0.125
Monthly per capita income				
< 2000	8.500 (0.609–118.637)	0.112		
2000–5000	9.000 (0.762–106.330)	0.081		
5000–10,000	9.286 (0.786–109.664)	0.077		
10,000–20,000	2.667 (0.208–34.197)	0.451		
> 20,000	ref			
Number of children	1.033 (0.611–1.747)	0.902		
Cause of Child's ECMO application				
Acute Myocarditis	ref			
Acute Respiratory Distress Syndrome	0.792 (0.347–1.807)	0.579		
Post-Cardiac Surgery	1.542 (0.529–4.493)	0.428		
Other	0.571 (0.192–1.700)	0.314		
Medical insurance				
Yes	0.715 (0.197–2.593-)	0.610		
No	ref			

**Figure 1:**
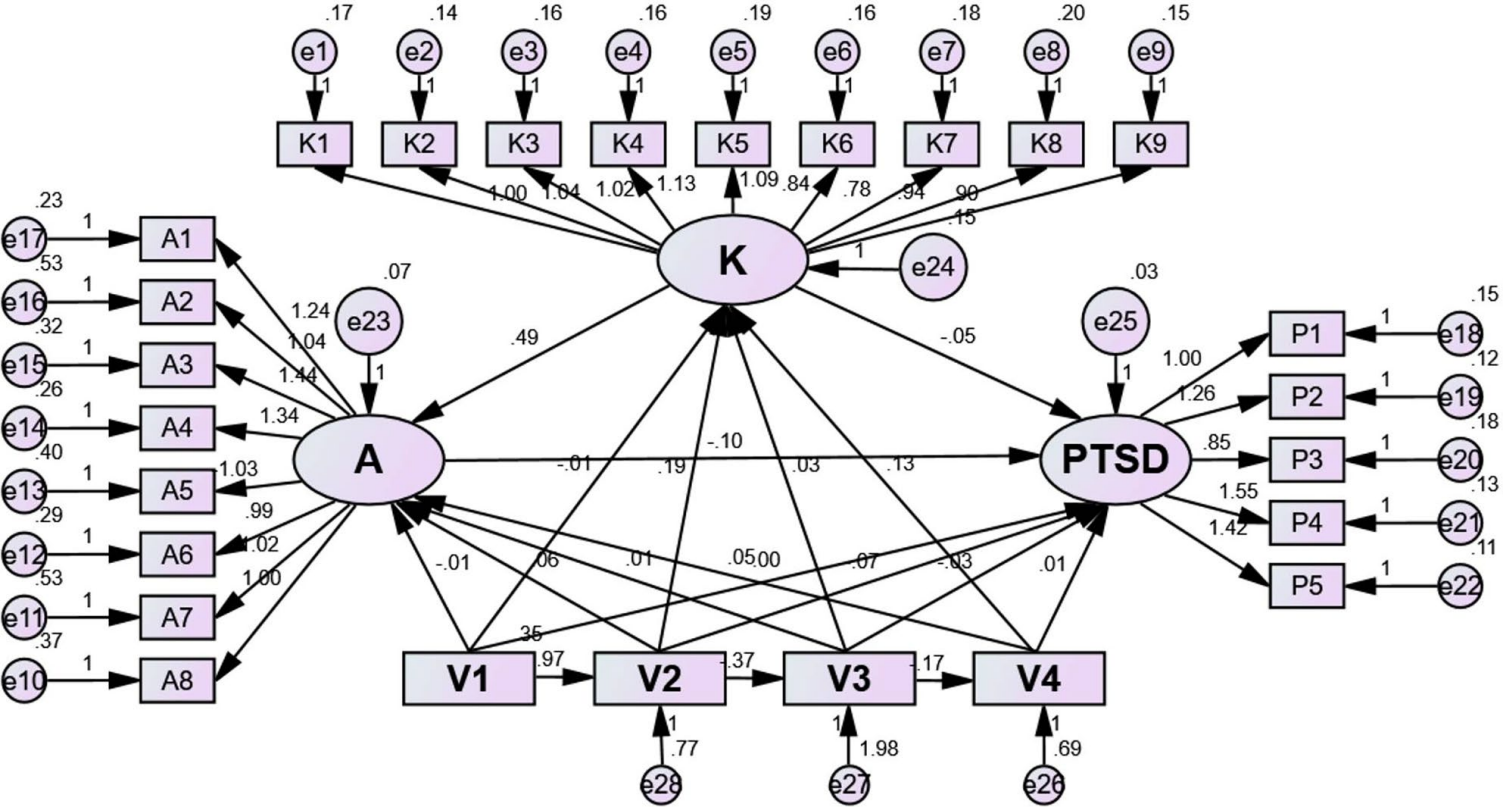
SEM for knowledge, attitude, and PTSS. Note: V1: residence; V2: education; v3: employment status; V4: monthly income.

Structural equation modeling (SEM) results provided insights into the relationships among variables. Residence had a direct impact on education (β = 0.973, P < 0.001), and education, in turn, directly influenced employment status (β = −0.372, P < 0.001), knowledge (β = 0.189, P < 0.001), attitude (β = 0.060, P = 0.047), and PTSS (β = −0.069, P = 0.003). Additionally, employment status exhibited direct effects on monthly income (β = −0.173, P < 0.001) and PTSS (β = −0.030, P = 0.017). Lastly, monthly income had a direct impact on knowledge (β = 0.130, P < 0.001), and knowledge, in turn, directly influenced attitude (β = 0.490, P < 0.001) (Fig. 1 and Supplementary Table 1).

## Discussion

Parents of children undergoing ECMO treatment demonstrated insufficient knowledge, suboptimal attitudes, and were affected by PTSS. This study underscores the necessity for tailored educational initiatives aimed at parents with lower educational backgrounds. Furthermore, it emphasizes the importance of implementing psychosocial support programs to address parental PTSS during ECMO treatment. Healthcare providers should also take into account socioeconomic factors when developing communication and support strategies to enhance the overall experience and outcomes of parents involved in ECMO care.

In this study, the examination of parents of children undergoing ECMO revealed inadequate knowledge, suboptimal attitudes, and elevated levels of PTSS symptoms. The findings of this study are consistent with previous research, indicating that family members of patients undergoing ECMO treatment in the Intensive Care Unit (ICU) consistently experience stress^12^. This may be attributed to their insufficient knowledge and suboptimal attitudes towards PTSS, particularly when the patient is a child. These outcomes underscore the imperative to enhance clinical practices in ECMO care^15^. Effective strategies should prioritize educational initiatives and improved communication to bolster parental understanding and foster positive attitudes^16^. Additionally, integrating psychological support mechanisms to address PTSS symptoms is crucial^17^. Implementing these comprehensive approaches will enable healthcare teams to deliver more holistic and supportive care to families navigating the challenges of pediatric ECMO treatment.

The study results underscore the critical role of demographic factors in influencing the experiences of parents with children undergoing ECMO. These findings suggest that a one-size-fits-all approach to ECMO care and support is inadequate. Gender disparities, along with variations associated with residence, education, employment status, and income, point to the need for tailored interventions to address the unique needs and challenges of different parental groups^18^. Therefore, to enhance clinical practice, it is imperative to develop and implement interventions that consider these demographic factors as key determinants in delivering empathetic and effective care for parents facing the challenges of pediatric ECMO^19^. Such tailored approaches can contribute to improved emotional well-being and overall outcomes for these families^20^.

The study revealed a notable deficiency in participants' knowledge about ECMO, with less than 10% indicating a “Very familiar” level of understanding across all knowledge section questions. Questions concerning fundamental ECMO concepts received the highest recognition, while those addressing more nuanced aspects of ECMO care generated significant unfamiliarity among participants. This highlights the necessity for comprehensive educational programs aimed at bridging this knowledge gap among healthcare professionals^21^. Such programs should cover both foundational and specialized ECMO topics to promote better-informed clinical practice and ultimately enhance patient outcomes.

Patient attitudes towards ECMO revealed important considerations for enhancing clinical practice. The generally positive outlook on ECMO's potential benefits and extended treatment time underscores the importance of comprehensive patient education, emphasizing both its advantages and limitations^18,22^. Patients' willingness to consider ECMO as a viable option despite its cost highlights the necessity of transparent discussions about healthcare expenses and financial considerations^23^. However, concerns about potential harm following ECMO application call for improved pre-ECMO counseling and support. These findings emphasize the pivotal role of communication and patient-centered care in refining ECMO clinical practices, ensuring they align with patients' preferences and needs^24–26^.

The analysis revealed significant relationships between key variables. Knowledge and attitude were positively correlated, indicating that as knowledge increases, attitudes tend to become more positive^27^. Conversely, negative correlations were observed between knowledge and PTSS, as well as between attitude and PTSS, suggesting that parents with more knowledge and positive attitudes may experience lower levels of PTSS^28,29^. SEM results showed that factors like residence influenced education, which in turn affected employment status, knowledge, attitude, and PTSS^30^. Employment status had direct effects on monthly income and PTSS. Additionally, monthly income influenced knowledge, which in turn affected attitude. These findings highlight the interconnectedness of various factors in shaping parental experiences and attitudes regarding pediatric ECMO, emphasizing the need for a comprehensive and tailored approach to support and education for parents^31,32^.

### Theoretical implications

The findings of this study contribute to the theoretical understanding of parental experiences and responses to stress in the context of pediatric medical interventions, specifically ECMO treatment. By identifying associations between education levels, employment status, and PTSS among parents, this research underscores the importance of socio-demographic factors in shaping parental psychological well-being. Additionally, the observed relationships between knowledge, attitude, and PTSS highlight the potential pathways through which parental understanding and perception of medical procedures may influence their emotional responses. These theoretical insights provide a basis for further research into the mechanisms underlying parental adaptation to pediatric medical crises and inform the development of targeted interventions to support parental coping and adjustment.

### Empirical implications

The empirical findings of this study offer valuable insights into the knowledge, attitude, and PTSS among parents of children undergoing ECMO treatment. The identification of specific demographic factors associated with elevated PTSS, such as lower education levels and occupation as housewives, provides empirical evidence for the differential impact of socio-economic status on parental psychological outcomes in this population. Moreover, the application of logistic regression and structural equation modeling techniques enhances our understanding of the complex interplay between socio-demographic variables, parental perceptions, and psychological distress. These empirical findings contribute to the evidence base for tailored interventions aimed at improving parental well-being and family functioning during pediatric medical crises.

### Limitations

This study had some limitations. First, the study's cross-sectional design limits our ability to establish causality or track changes in knowledge, attitude, and PTSS over time^33^. Longitudinal research could provide a more comprehensive understanding of how these factors evolve during the ECMO experience. Second, the data collection method, which relied on a web-based survey, may introduce selection bias as it excludes individuals without internet access or those who might not have participated due to various reasons. Additionally, self-report questionnaires are subject to response bias, as participants may provide socially desirable responses or recall inaccuracies.

### Recommendations

Based on the findings and limitations of this study, several recommendations emerge for future research and clinical practice. Longitudinal studies employing mixed-methods approaches are warranted to investigate the longitudinal trajectories of parental adjustment and identify modifiable factors contributing to resilience or vulnerability. Clinically, healthcare professionals should implement routine screening for parental PTSS and provide accessible psychosocial support services tailored to the needs of parents, particularly those with lower education levels or limited social support networks. Educational interventions aimed at enhancing parental knowledge and fostering positive attitudes towards medical procedures should be integrated into the ECMO care protocol to empower parents and alleviate distress. Collaborative efforts between researchers, healthcare providers, and policymakers are essential to address the multifaceted needs of parents facing pediatric medical crises and promote optimal family-centered care.

In conclusion, parents of children undergoing ECMO often lack sufficient knowledge, exhibit suboptimal attitudes, and may experience PTSS. To enhance their experience, we recommend comprehensive education, with a focus on parents with lower education levels. Addressing parental PTSS through psychosocial support and regular screening is crucial. Integrating socioeconomic factors into support strategies can tailor care to individual needs, leading to improved outcomes for parents and their children during ECMO treatment.

### Supplementary Information

Supplementary Information.

## Data Availability

All data generated or analysed during this study are included in this published article and its supplementary information files.
