# Supplementary material for: Parents' knowledge and attitudes towards extracorporeal membrane oxygenation and their post-traumatic stress symptoms
Source: Sci Rep. 2024 May 10;14:10700. doi: 10.1038/s41598-024-60880-3 (PMC11087580; doi:10.1038/s41598-024-60880-3)
Supplement: Supplementary file 1 — Supplementary Information. [file 41598_2024_60880_MOESM1_ESM.docx]

**Supplement Table 1 SEM Results**

|  |  |  | Estimate | S.E. | C.R. | P |
| --- | --- | --- | --- | --- | --- | --- |
| V2 | <--- | V1 | .973 | .105 | 9.262 | <0.001 |
| V3 | <--- | V2 | -.372 | .095 | -3.922 | <0.001 |
| V4 | <--- | V3 | -.173 | .040 | -4.300 | <0.001 |
| K | <--- | V1 | -.011 | .058 | -.190 | .849 |
| K | <--- | V2 | .189 | .036 | 5.244 | <0.001 |
| K | <--- | V3 | .033 | .022 | 1.511 | .131 |
| K | <--- | V4 | .130 | .036 | 3.629 | <0.001 |
| A | <--- | V1 | -.007 | .046 | -.156 | .876 |
| A | <--- | V2 | .060 | .030 | 1.983 | .047 |
| A | <--- | K | .490 | .093 | 5.280 | <0.001 |
| A | <--- | V3 | .014 | .017 | .799 | .424 |
| A | <--- | V4 | .048 | .029 | 1.649 | .099 |
| PTSD | <--- | A | -.097 | .076 | -1.278 | .201 |
| PTSD | <--- | K | -.047 | .059 | -.803 | .422 |
| PTSD | <--- | V4 | .006 | .020 | .291 | .771 |
| PTSD | <--- | V3 | -.030 | .013 | -2.386 | .017 |
| PTSD | <--- | V2 | -.069 | .023 | -3.021 | .003 |
| PTSD | <--- | V1 | -.001 | .032 | -.037 | .971 |
| K1 | <--- | K | 1.000 |  |  |  |
| K2 | <--- | K | 1.044 | .096 | 10.858 | <0.001 |
| K3 | <--- | K | 1.022 | .098 | 10.456 | <0.001 |
| K4 | <--- | K | 1.125 | .103 | 10.886 | <0.001 |
| K5 | <--- | K | 1.089 | .105 | 10.419 | <0.001 |
| K6 | <--- | K | .837 | .088 | 9.463 | <0.001 |
| K7 | <--- | K | .781 | .089 | 8.771 | <0.001 |
| K8 | <--- | K | .942 | .099 | 9.541 | <0.001 |
| K9 | <--- | K | .898 | .090 | 9.929 | <0.001 |
| A8 | <--- | A | 1.000 |  |  |  |
| A7 | <--- | A | 1.016 | .201 | 5.063 | <0.001 |
| A6 | <--- | A | .987 | .171 | 5.773 | <0.001 |
| A5 | <--- | A | -1.028 | .187 | -5.487 | <0.001 |
| A4 | <--- | A | 1.343 | .204 | 6.578 | <0.001 |
| A3 | <--- | A | 1.439 | .222 | 6.490 | <0.001 |
| A2 | <--- | A | 1.042 | .204 | 5.119 | <0.001 |
| A1 | <--- | A | 1.235 | .189 | 6.525 | <0.001 |
| P1 | <--- | PTSD | 1.000 |  |  |  |
| P2 | <--- | PTSD | 1.263 | .244 | 5.175 | <0.001 |
| P3 | <--- | PTSD | .849 | .213 | 3.982 | <0.001 |
| P4 | <--- | PTSD | 1.550 | .287 | 5.394 | <0.001 |
| P5 | <--- | PTSD | 1.425 | .263 | 5.413 | <0.001 |
